# Indirect shoot organogenesis from leaf explants of *Adhatoda vasica* Nees

**DOI:** 10.1186/2193-1801-3-648

**Published:** 2014-11-03

**Authors:** Jaydip Mandal, Undurthy Laxminarayana

**Affiliations:** Department of Education in Science and Mathematics, Regional Institute of Education, National Council of Educational Research and Training, Shyamla Hills, Bhopal, 462013 India; Department of Education, Regional Institute of Education, National Council of Educational Research and Training, Mysore, 570006 India

**Keywords:** *Adhatoda vasica*, Adventitious shoots, Callus induction, Organogenesis, Shoot regeneration

## Abstract

A novel protocol for indirect shoot organogenesis of *Adhatoda vasica* was developed using petiole explants derived from mature shrubby plants. Media with concentrations of cytokinins in combination with auxins were used to induce callus formation in two explants types: petiole and leaf segment. The frequency of callus formation from petiole and leaf segment explants on Murashige and Skoog (MS) basal medium supplemented with 0.25 mg l^−1^ thidiazuron (TDZ) and 0.25 mg l^−1^ α-naphthaleneacetic acid (NAA) was 100 ± 0.0 and 83.70 ± 0.52% respectively, while on this medium supplemented with 0.25 mg l^−1^ 6-(γ-γ, dimethylallyamino purine) (2iP) and 0.25 mg l^−1^ NAA, the callus frequency was 100 ± 0.0 and 96.70 ± 0.67% respectively. The highest shoot regeneration (90.60 ± 0.52%) response and the maximum shoots (8.10 ± 0.28) per callus were achieved from petiole explants on MS medium containing 0.25 mg l^−1^ TDZ and 0.25 mg l^−1^ NAA. On the contrary, on Schenk & Hildebrandt (SH) basal medium supplemented with 0.25 mg l^−1^ TDZ and 0.25 mg l^−1^ NAA, the frequency of callus formation from petiole and leaf segment explants was 100 ± 0.0 and 90.50 ± 0.89% respectively while the callus frequency on this medium containing 0.25 mg l^−1^ 2iP and 0.25 mg l^−1^ NAA was 100 ± 0.0 and 89.90 ± 0.72% respectively. The shoot regeneration frequency for petiole explants was 89.90 ± 0.46% producing 6.00 ± 0.21 shoots per callus on SH basal medium supplemented with 0.25 mg l^−1^ TDZ and 0.25 mg l^−1^ NAA. Whereas petiole explants could induce 83.70 ± 0.50% shoot regeneration and 7.3 ± 1.05 shoots per callus on SH medium containing 0.25 mg l^−1^ indole-3-butyric acid (IBA), 0.5 mg l^−1^ 6-benzyladenine (BA) and 0.5 mg l^−1^ 2iP. Elongation of regenerated shoot was obtained on MS basal medium supplemented with 0.25 mg l^−1^ TDZ. All regenerated shoots developed adventitious roots within 4 weeks when transferred to rooting medium containing SH medium supplemented with 0.5 mg l^−1^ IBA. Total nine rooted plantlets were transferred from in vitro to in vivo conditions and eight plants survived and successfully acclimatized in the shaded greenhouse 12 weeks after transplanting.

## Introduction

The *Adhatoda vasica* Nees. belonging to the genus *Adhatoda* in the family Acanthaceae, is one of the important shrub species. This medicinal shrub which is native to India and has been naturalized in other parts of the world such as the tropical regions of Southeast Asia enjoys wide reputation for its novelty of potential medicinal uses. The leaves, the roots and flowers of *Adhatoda vasica* are extensively used in indigenous medicine especially as bronchodilator, antidiabetic, anti-jaundice and expectorant among many medicinal uses (Maurya and Singh 2010). There are many reports which highlight the efficacy of the alkaloid vasicine and its semi synthetic derivatives such as bromohexine and ambroxol is exhibited in its inhibitory effects against *Mycobacterium tuberculosis* (Grange and Snell 1996). This medicinal plant possesses certain chemicals such as alkaloids, phenolics, flavonoids and sterols which have diverse medicinal applications including cardiovascular protection, abortifacient, antitubercular, antimutagenic, antiulcer, antiallergic, oxitocic, anti-inflammatory, antiasthmatic, hepatoprotective and antitussive activities (Singh et al. 2011). This species is receiving increasing attention as it is commonly used in indigenous and traditional folk medicine system in South-East Asia. There are many reports which highlight the medicinal uses of this shrub such as alkaloids vasicine and vasicinone from leaves and roots as antidiabetic, bronchodilator, expectorant, antispasmodic and oxitocic activity (Dinesh and Parameswaran 2009) besides having potential to inhibit human immunodeficiency virus (HIV) (Nath and Buragohain 2005). The oil from leaves, flowers and roots of *Adhatoda vasica* plant exhibits high activity against tubercle bacilli (*Mycobacterium tuberculosis*) (Grange and Snell 1996). The leaf extract shows chemopreventive efficacy especially stimulative for liver, lung and forestomach (Singh et al. 2000). Moreover, plant-derived medicines such as reserpine (antihypertensive), digoxin (cardiotonic), vinblastine and paclitaxel (antineoplasic), morphine (analgesic), codeine (antitussive), artimisinin (antimalarial) etc. constitute potential health care systems (Gomez-Galera et al. 2007). Besides, biochemical constituents of extracts from different parts of medicinal plants are subject to alteration and vary in concentration depending on the physicochemical properties of species that directly or indirectly in communion with environmental factors (Murch et al. 2000). The advent of Plant Biotechnology has ushered in the production and commercialization of bioengineered (modified) plants with the inbuilt capacity to expressing novel genes conferring beneficial effects on food security, human health care, the environment and conservation of biodiversity (Vasil 2008).

The propagation of *Adhatoda vasica* is restricted due to poor seed sets, low potential for seed germination and through shoot cuttings which solely rely on season for multiplication (Anand and Bansal 2002; Maurya and Singh 2010). In vitro propagation through axillary shoot proliferation is a useful technique for producing clonal plantlets while shoot regeneration via adventitious bud induction is a promising tool in order to explore variability, to bring forth new characteristics of agronomic value and to develop new varieties through genetic transformation (Corredoira et al. 2008). In vitro studies for shoot regeneration of *Adhatoda vasica* using nodal segment and shoot tip explants (Abhyankar and Reddy 2007; Nath and Buragohain 2005; Khalekuzzaman et al. 2008) have been reported. In other species of the family Acanthaceae, successful multiple shoot initiation and shoot regeneration were achieved from split nodal halve of *Adhatoda beddomei* (Sudha and Seeni 1994) and mature explants of *Beloperone plumbaginifolia* (Shameer et al. 2009). Successful adventitious bud regeneration in *Adhatoda vasica* was limited to reports of shoot formation from callus through internode segments of mature plants (Azad and Amin 1998) and young leaves and cotyledon explants (Amin et al. 1997; Azad et al. 1999).

Therefore, in present study an efficient regeneration system is devised for a rapid propagation in *Adhatoda vasica* through callus mediated induction of adventitious buds and shoots in leaf explants derived from mature plant. Consequently, it will meet the pharmaceutical requirements besides strengthening solidarity to the increasing concern to conserve biodiversity through preservation of germplasm of this medicinal shrub. In addition, this protocol of shoot bud regeneration system of *Adhatoda vasica* could provide the target organs for use in gene transformation experiments for further improvement of proven value genotypes.

## Materials and methods

### Plant material and ex vitro derived leaves

Mature plants of *A.vasica* growing on the experimental garden of Regional Institute of Education campus were used as explants sources. The first formed 7–10 day old leaves of 9.0 cm × 2.0 cm length from a single genotype were collected on November 1^st^ 2010 and surface disinfested by immersing in 0.1% HgCl_2_ (w/v) with constant stirring for 5 min and then rinsing five times, each of 2 min with sterile double distilled water under aseptic condition. Explants were prepared by transverse cut with a sterile surgical blade to petiole explant of 2.0 cm × 2.0 cm and leaf segment of 2.0 cm × 1.0 cm.

### Callus induction and maintenance

Two different basal media were used to induce callus formation: MS (Murashige and Skoog 1962) and SH (Schenk and Hildebrandt 1972). Each excised explant was incubated into 100 ml conical flasks (Borosil, India) containing 20 ml medium supplemented with various combinations of auxins and cytokinins. The medium was supplemented with: 0.25 or 1.0 mg l^−1^ indole-3-butyric acid (IBA), α-naphthaleneacetic acid (NAA) or 2,4-dichlorophenoxyacetic acid (2,4-D) in combination with: 0.25 or 1.0 mg l^−1^ 6-benzyladenine (BA), thidiazuron (TDZ) or 6-(γ-γ, dimethylallyamino purine)(2iP). The pH of the medium was adjusted to pH 5.8 with 1 N NaOH and 0.1 N HCl prior to the addition of 7.5 g l^−1^ agar, the gelling agent and autoclaved at 121°C and 103.5 kPa for 20 min. Each experiment consisted of 5 conical flasks per treatment and 4 explants of each type: LSWP and LS per flask. All the explants were cultured on the media in a growth chamber at 25°C under 16 h light/8 h dark photoperiod provided by white fluorescent tubes at 35 μmol m^−2^ s^−1^. Leaf cultures were maintained on the treatment medium for 4 weeks in a growth room at the same culture conditions. Callus induced at the petiole explants and around the cut surface of leaf segment explants was excised (2–3 mm diameter pieces) with scalpel after 4 weeks of inoculation and transferred to treatments for induction of organogenic callus. Effect of various auxin/cytokinin combinations on callus induction was evaluated based on the appearance and consistence of callus formation in each treatment.

### Shoot differentiation and proliferation

After 4 weeks of callus induction, only well developed regenerating calli (2–3 mm diameter pieces) were transferred to two basal media: Murashige and Skoog (MS) medium and Schenk & Hildebrandt (SH) medium and supplemented with 0.25 mg l^−1^ NAA or IBA in combination with 0.25 mg l^−1^ BA, 2iP or TDZ. The regenerating callus cultures were maintained in growth chambers on the same medium with controlled conditions (25°C and 16 h light and 8 h dark photoperiod) for 8 weeks for shoot induction. There were four replicate culture flasks with 5 explants (callus pieces of 1.0-2.5 g fwt) each per treatment and the experiment was repeated twice. The number of well developed shoots regenerated per callus was counted after 4 weeks of incubation. Regenerated shoots were placed onto MS medium supplemented with 0.25 mg l^−1^ TDZ and incubated for 4 weeks to initiate shoot elongation and proliferation of multiple shoots. The adventitious shoots were subcultured on the same culture medium every 3 weeks.

### In vitro rooting and acclimatization

One to two cm long shoots were isolated from shoot clusters of elongation medium and transferred individually to SH medium supplemented with IBA or NAA (0.5 or 1.0 mg l^−1^) and incubated for 4 weeks for root development. After 4 weeks of incubation in light the rooted shoots were carefully removed from culture vessels and washed in running tap water to free agar. Healthy rooted shoots were transplanted to garden soil in plastic pots and kept jacketed with polyethylene bags in growth room under the same culture conditions as for shoot differentiation. The plantlets were maintained under high humidity for 8 weeks by spraying with liquid ½ strength MS in every alternate day and slowly weaned to lower humidity by making small holes around the polyethylene bags of potted plantlets for a period of further 12 weeks. All the surviving plants (90%) were transferred to green house with removal of polyethylene bag before exposure to the experimental field under natural photoperiod of 9–11 h and a temperature of 20-29°C.

### Statistical analysis

All the experiments described above were conducted in a completely randomized design and repeated twice. Means and standard error of means for all dependent variables such as callus induction, shoot regeneration, shoot number and shoot length under different plant growth regulator concentrations were computed and determined the significant differences between means using Tukey test.

## Results

**Figure 1:**
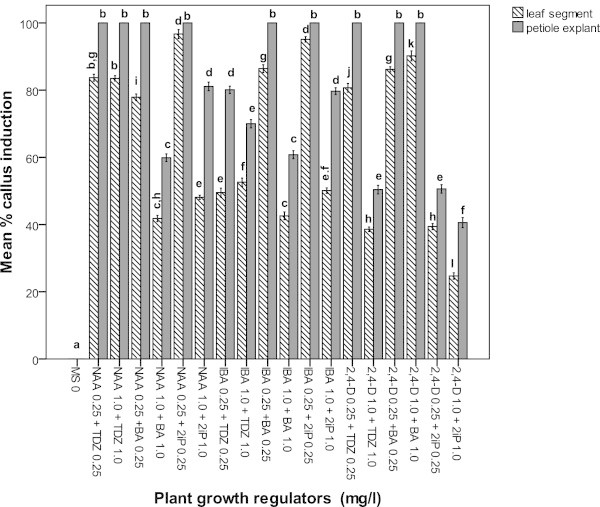
**Mean % of callus induction of leaf segment and petiole explants of** ***Adhatoda vasica*** **on MS medium containing plant growth regulators after 4 weeks.** Different letter(s) indicate a significant difference between treatments at *P ≤*0.05 according to Tukey test.

**Figure 2:**
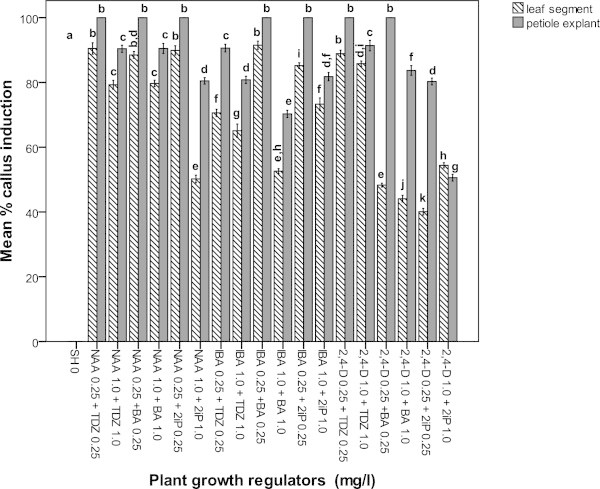
**Mean % of callus induction of leaf segment and petiole explants of** ***Adhatoda vasica*** **on SH medium containing plant growth regulators after 4 weeks.** Different letter(s) indicate a significant difference between treatments at *P ≤*0.05 according to Tukey test.

**Figure 3:**
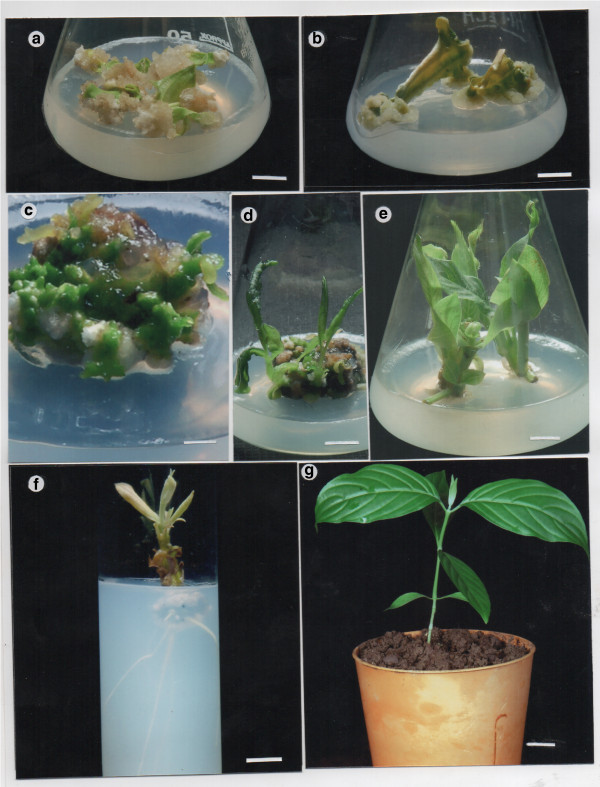
**a-g Adventitious shoot regeneration of** ***Adhatoda vasica*** **. a** Friable callus formation from petiole explants after 3 weeks of culture on MS medium supplemented with 0.25 mg l ^−1^ 2,4-D and 0.25 mg l ^−1^ TDZ. **b** Compact callus formation from petiole explants on MS medium supplemented with 0.25 mg l ^−1^ NAA and 0.25 mg l ^−1^ TDZ after 3 weeks of culture on the same medium. **c** Onset of organogenesis on MS medium supplemented with 0.25 mg l ^−1^ NAA and 0.25 mg l ^−1^ TDZ 4 weeks after culture initiation. **d** Adventitious multiple shoots on the same medium after 8 weeks of culture initiation. **e** Elongation growth of shoots on MS medium supplemented with 0.25 mg l ^−1^ TDZ after 2 weeks of culture. **f** Rooting of in vitro shoot on MS medium supplemented with 0.5 mg l^−1^ IBA after 4 weeks of culture. **g** Acclimatized plant after 8 weeks of transfer to garden soil. Bars =1 cm **(a and b)**, 3 cm **(c)**, 1 cm **(d and e)**, 3 cm **(f)**, and 1 cm **(g)**.

Incubation of petiole and leaf segment explants of *Adhatoda vasica* either on MS or SH medium supplemented with different concentrations of NAA, IBA or 2,4-D (0.25 or 1.0 mg l^−1^) in combination with TDZ, BA or 2iP (0.25 or 1.0 mg l^−1^) produced callus within 2 weeks (Figures 1 and 2). Callus primarily originated in petiole explants whereas callus development was observed on cut off surface of leaf segment explants. The explants cultured on the control medium (MS or SH medium) exhibited no callusing response. MS medium produced a significantly higher callusing from both petiole and leaf segment explants than on SH medium irrespective of concentrations and combinations of hormones used (Figures 1 and 2). The callus initiation response (100 ± 0%) from petiole explants was observed on MS medium supplemented with NAA, or IBA or 2,4-D (0.25 or 1.0 mg l^−1^) in combination with TDZ or BA or 2iP (0.25 or 1.0 mg l^−1^). On the other hand, the leaf segment explants induced the maximum callus formation (100 ± 0%) when NAA, or IBA (0.25 mg l^−1^) was added to the MS medium containing 2iP (0.25 mg l^−1^) after 4 weeks of culture. The petiole explant was found to be better explant source for callus induction than leaf segment explants, as the former produced higher percentage of callus than the latter (Figures 1 and 2). MS medium containing either 0.25 or 1.0 mg l^−1^ NAA in combination with 0.25 or 1.0 mg l^−1^ TDZ or BA or 2iP produced green and compact callus from both petiole and leaf segment explants whereas friable callus was induced on MS medium containing either IBA or 2,4-D in combination with TDZ or BA or 2iP at the same level of concentrations (Figures 1 and 3a, b). In contrast, among the different concentrations and combinations of plant growth regulators tested, the best callus induction from petiole explants (100 ± 0%) was achieved when the SH medium was supplemented with 0.25 mg l^−1^ NAA in combination with TDZ or BA or 2iP while leaf segment explants produced the highest callus (88.50 ± 0.52 to 90.50 ± 0.89%) at the same level of concentrations after 4 weeks of culture (Figure 2). Petiole explants showed best callus induction (100 ± 0%) on SH medium containing either 0.25 mg l^−1^ IBA in combination with 0.25 mg l^−1^ BA or 2iP and 0.25 mg l^−1^ 2,4-D in combination with 0.25 mg l^−1^ TDZ or BA. Leaf segment explants, in comparison, showed best callus induction in the range of 85.20 ± 0.44 to 91.50 ± 0.62% and 48.30 ± 0.30 to 88.90 ± 0.53% at the same level of concentrations (Figure 2).

### Regenerating callus and shoot regeneration

**Figure 4:**
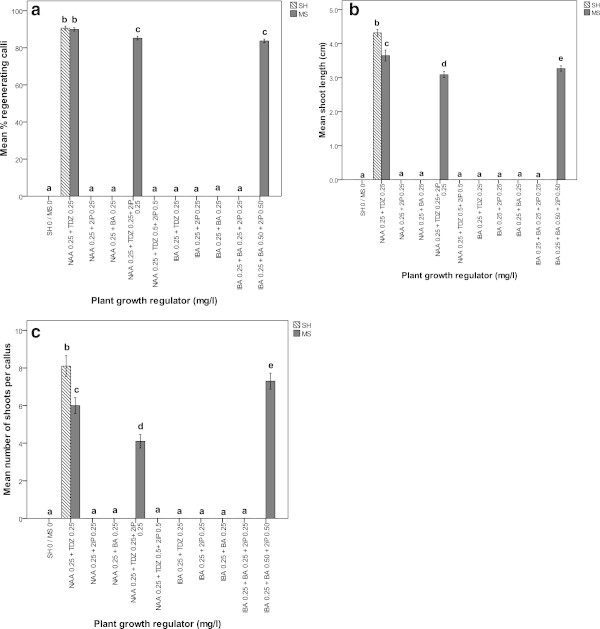
**a-c Organogenic responses of petiole explants of** ***Adhatoda vasica*** **on MS and SH medium supplemented with plant growth regulators after 4 weeks. a)** Mean % regenerating calli. **b)** Mean number of shoots per callus. **c)** Mean shoot length (cm). Different letter(s) indicate a significant difference between treatments at *P* ≤0.05 according to Tukey test.

After a 2-subculture, each of 14 days on respective callus inducing medium which contained NAA, IBA or 2,4-D (0.25 or 1.0 mg l^−1^) in combination with TDZ, BA or 2iP (0.25 or 1.0 mg l^−1^), the callus derived from petiole and leaf segment explants were cut into pieces (2–3 mm diameter pieces) and transferred onto either MS or SH medium supplemented with 0.25 or 1.0 mg l^−1^ NAA or IBA in combination with 0.25 or 1.0 mg l^−1^ TDZ or 2iP or BA for induction of regenerating callus. During the induction stage, callus derived from petiole explants was observed efficiently conversion to regenerating callus either on MS or SH medium containing plant growth regulators while regenerating callus could not be induced from callus derived from leaf segment explants even after a 6-month subcultures. The highest frequency of regenerating callus (90.60 ± 0.52%) response and the maxium number of shoots per callus explants (8.10 ± 0.28) (significantly different P <0.05) with maxium shoot length (4.32 ± 0.05 cm) were achieved on MS medium supplemented with 0.25 mg l^−1^ NAA and 0.25 mg l^−1^ TDZ (Figures 3c, d and 4a-c). In comparison, induction of regenerating callus and its conversion to shoots were observed on SH medium supplemented with either NAA in combination with TDZ or NAA and TDZ in combination with 2iP or IBA and BA in combination with 2iP (Figure 4a and b). In contrast, the highest percentage of regenerating callus (89.90 ± 0.46%) from petiole explants induced the maximum number of shoots per callus (6.00 ± 0.21) with an average shoot length of 3.65 ± 0.08 cm on SH medium containing 0.25 mg l^−1^ NAA and 0.25 mg l^−1^ TDZ. Besides, the addition of 0.25 mg l^−1^ 2iP to SH culture medium containing 0.25 mg l^−1^ NAA and 0.25 mg l^−1^ TDZ stimulated regenerating callus rate (85.20 ± 0.51%) and induced the maximum number of shoots per callus explants (4.10 ± 0.18) with an average shoot length of 3.09 ± 0.05 cm within 4 weeks of culture. The addition of higher concentration of 2iP (0.5 mg l^−1^) in plant regenerating SH medium containing 0.5 mg l^−1^ BA and 0.25 mg l^−1^ IBA induced conversion of regenerating callus (83.70 ± 0.50%) into the maximum number of shoots per callus explant (7.3 ± 1.05) with an average shoot length of 3.27 ± 0.04 cm (Figure 4a-c). The adventitious shoots produced via indirect organogenesis were isolated from callus explants and incubated on MS medium supplemented with 0.25 mg l^−1^ TDZ for elongation growth (Figure 3e). The shoots with well developed leaves were subcultured on this elongation growth medium in every three weeks.

### Rooting and acclimatization

After 4 weeks of incubation on the SH medium supplemented with 0.5 mg l^−1^ IBA regenerated shoots obtained from petiole explants produced 9–10 healthy roots per shoot with 75% rooting (Figure 3f). Total nine rooted plantlets were transferred from in vitro to in vivo conditions and eight plants were successfully acclimatized for 12 weeks in a garden soil under green house conditions. The rooted plantlet had high survival rate (90%) (data not shown). The plantlet grew successfully into normal plant (Figure 3g).

## Discussion

Calli were obtained from all the explants types: petiole explants produced the highest callusing percentage compared to leaf segment explants. This suggests that the source of explants with varying endogenous auxin concentrations or their hormone responsivity might play a role in determining callus induction ability (Lane 1978). Higher callusing response on MS medium than SH medium was possibly attributable to the differences in nitrogen source which may play a critical role in callus induction and morphogenesis (George 1993). Preliminary investigation of callus induction, organogenic callus formation and shoot regeneration potential of both leaf segments and petiole explants revealed that all explants types induced callus but only petiole explants derived callus was responsive to organogenic callus formation and shoot regeneration. These results are consistent with previous reports that callus derived from explants of varying potentials results in different morphogenic responses (Erisen et al. 2010; Ghimire et al. 2010; Piovan et al. 2010; Sun and Hong 2010; Wadl et al. 2011; Shaik et al. 2011; Lin et al. 2011; Kumar and Thomas 2012). In the present study, the highest number of shoot regeneration (8.10 ± 0.28) in *Adhatoda vasica* was obtained from petiole explants on MS medium supplemented with 0.25 mg l^−1^ NAA and 0.25 mg l^−1^ TDZ within 4 weeks of incubation of culture. Similarly, the positive effect of BA or Kin or TDZ or 2iP or Zn in combination with IAA, or NAA on in vitro shoot regeneration through organogenic differentiation has been reported by several researchers (Bhagya et al. 2013; Erisen et al. 2010; Unda et al. 2007; Lin et al. 2011; Mandal and Laxminarayana 2012; Perez-Jimenez et al. 2012; Wadl et al. 2011; Ghimire et al. 2010; Shen et al. 2007). In comparison, though shoot organogenesis was observed on SH medium supplemented with NAA and TDZ combination, optimum induction of shoot regeneration in the petiole derived callus required the addition of 2iP either on SH medium containing NAA and TDZ or 2iP in combination with IBA and BA for maximum shoot regeneration frequency (83.70 ± 0.50%) and adventitious in vitro shoot regeneration (7.3 ± 1.05) whereas shoot organogenesis was not observed on MS medium with these combinations of plant growth regulators. The highest shoot production (8.10 ± 0.28), in the present study, on MS medium containing NAA and TDZ was consistent with the previous reports of Ghimire et al. (2012), Korban et al. (1992) and Nabors et al. (1983) where TDZ and NAA have been more widely used for regeneration of adventitious shoots than other cytokinins and auxins respectively. Similarly, adventitious shoot buds were induced from young leaf explants of *Jatropha curcas* on MS medium supplemented with TDZ, BA and IBA (Deore and Johnson 2008) and from mature leaf tissues of *Pelargonium capitatum* on MS medium supplemented with TDZ, BA and NAA (Arshad et al. 2012). Likewise in many shoot multiplication studies, 2iP was crucial as potential cytokinin for shoot formation, shoot multiplication and plant regeneration (Voyiatzi and Voyiatzis 1989; Shen et al. 2007; Mallon et al. 2011). It may be highlighted that cytokinins not only contribute to maintaining a small population of stem cells that continuously generates organs and tissues but also mediates light responses of stem cells that lead to initiation of primordia, the progenitors of new organs. Besides, a high local auxin concentration as a major signal redirects the cytokinin stimulated growth to new organ initiation and positioning (Murray et al. 2012). In the present study, rooting of in vitro shoots of *Adhatoda vasica* developed readily on SH medium containing IBA which was similar to the reports of Erisen et al. (2010) who achieved optimum number roots of in vitro shoot cuttings of *Austragalus cariensis* on MS medium supplemented with IBA.

In *Adhatoda vasica*, callus derived from mature explants of petiole produced adventitious buds both on MS medium supplemented with NAA and TDZ and SH medium either supplemented with NAA and TDZ or with 2iP, NAA and TDZ or with 2iP, IBA and BA. Although, it was reported that callus derived from mature explants was not amenable to regeneration (Amin et al. 1997; Azad et al. 1999), adventitious shoots were induced from callus derived from mature internode explants of this species on medium containing BA and NAA (Azad and Amin 1998).

In conclusion, this research study presents the first report of plant regeneration via organogenesis of *Adhatoda vasica* from petiole explants. Although both petiole and leaf segment explants produced callus, higher potential for organogenic differentiation from callus derived from petiole explants was observed on MS medium than on SH medium supplemented with 0.25 mg l^−1^ NAA and 0.25 mg l^−1^ TDZ. The protocol presented in this study may provide a high efficiency regeneration system for successful regeneration of adventitious shoots for ex situ preservation of *Adhatoda vasica* as well as genetic improvement studies for pharmaceutical uses and future research investigations.

## Abbreviations

BA: 6-benzyladenine
IBA: Indole-3-butyric acid
Kin: kinetin
2: 4-D: 2, 4-dichlorophenoxyacetic acid
2iP: 6-(γ-γ, dimethylallyamino purine)
NAA: α-naphthaleneacetic acid
TDZ: Thidiazuron
Zn: Zeatin.
